# The Effects of Essential Amino Acid Supplementation on Hippocampal Neurotrophin, Dopaminergic and Serotonergic Changes in an Overtraining Mouse Model

**DOI:** 10.3390/nu17182957

**Published:** 2025-09-16

**Authors:** Lior Binman, Tavor Ben-Zeev, Asher Harris, Chagai Levi, Inbal Weissman, David D. Church, Arny A. Ferrando, Jay R. Hoffman

**Affiliations:** 1 Sport Science Program, School of Health Science, Ariel University, Ariel 40700, Israel; 2 Center for Translational Research in Aging and Longevity, University of Arkansas for Medical Sciences, Little Rock, AK 72205, USA; dchurch@uams.edu (D.D.C.); aferrando@uams.edu (A.A.F.)

**Keywords:** BDNF, TrkB, dopamine, serotonin, resistance exercise

## Abstract

**Background/Objectives**: This study examined the efficacy of essential amino acid (EAA) supplementation on changes in behavior and hippocampal neurotrophin, dopaminergic and serotonergic markers to a volume overload stress resembling an overtraining syndrome. **Methods**: Thirty-two 3-month-old male C57Bl/6J mice were randomized into four groups: Resistance training (RT), resistance training with overtraining (RTO), resistance training with overtraining and EAA (RTOEAA), or control. Mice in RTOEAA received EAA supplementation (1.5 g·kg·day^−1^), while the other groups received a sham treatment. A 5-week resistance training protocol was employed. Training volume was increased two-fold during the final two weeks for RTO and RTOEAA to cause the OTS. EAA intervention for RTOEAA occurred during the OTS. **Results**: A significant decline in the maximum resistance carrying load in RTO compared to RT (*p* = 0.002) and RTOEAA (*p* = 0.029) confirmed that the animals in that group were overtrained. Significantly greater average latency times for RTO compared to RT (*p* = 0.009) and C (*p* = 0.05) indicated that the OTS caused spatial memory deficits in animals that were not supplemented. These latter changes may have been related to the significant declines in brain derived neurotrophic (BDNF) expression and elevations in dopamine 1 receptor (D1R) expressions. Increased resiliency for RTOEAA may have been related to the effect of EAA on stimulating significant increases in the expression of hippocampal tyrosine receptor kinase B (TrkB) and serotonin receptors (5-HT1A). **Conclusions**: EAA supplementation during a resistance model of overtraining appeared to provide increased resiliency to OTS by maintaining neurotrophin expression and enhancing serotonergic adaptation.

## 1. Introduction

Physical training programs stimulate the body’s adaptive mechanisms, enhancing cardiovascular, muscular, and metabolic performance [1]. However, excessive training without sufficient recovery can lead to maladaptive responses, resulting in performance decrements and behavioral changes. If these conditions persist, an overtraining syndrome (OTS) may result [2]. It has been estimated that between 20% and 60% of competitive endurance athletes will be impacted by OTS during their careers [3]. Common symptoms associated with OTS include persistent fatigue, lethargy, physical performance decrements, as well as mood disturbances such as depression, anxiety, irritability, and emotional instability [4].

The OTS extends beyond physical exhaustion and shares physiological stress responses with mood disorders such as depression and anxiety [5]. While OTS is not classified as a mental disorder, it involves the dysregulation of key stress-response systems, including the hypothalamic-pituitary axis (HPA) and neurotransmitter networks [5]. Significant elevations in circulating cortisol concentrations may be seen in anaerobic athletes that are overtraining, while decreases in circulating concentrations in cortisol concentrations may be noted in endurance athletes that are overtraining [5]. Research indicates that OTS not only diminishes physical performance but also leads to cognitive impairments and emotional disturbances, including reduced executive function and mood dysregulation [6,7]. Others have suggested that athletes with OTS experience worsened mood states, cognitive impairments, and heightened emotional reactivity due to hormonal (e.g., testosterone and estradiol) and metabolic dysfunction [8]. If unresolved, chronic OTS can lead to prolonged psychological distress, increased injury risk, and, in severe cases, premature retirement from competitive sport [8]. Rest and appropriately developed periodized training programs with adequate nutrition help relieve OTS symptoms but may not fully correct underlying neurobiological imbalances, requiring targeted non-pharmacological nutritional interventions [9]. The overlap between OTS and mental disorders suggests that they may share underlying neurobiological mechanisms [10].

Evidence indicates that OTS induces maladaptive effects on the endocrine system, particularly disrupting the HPA axis [11,12]. Prolonged dysregulation of the HPA axis in OTS disrupts cortisol homeostasis, weakens negative feedback inhibition, and alters glucocorticoid receptor expression in the hippocampus, contributing to cognitive impairments such as memory deficits and slowed processing speed [13,14]. HPA axis impairment is well-documented in both human and animal models of chronic stress, depression, and anxiety [15,16,17]. Elevated cortisol concentrations are commonly observed in mental disorders, such as depression and anxiety reflecting HPA axis dysregulation, which is consistent with observations in athletes with OTS [18]. Furthermore, prolonged exposure to stress is also associated with neurotransmitter imbalances, and impaired neuroplasticity [18]. Studies have demonstrated that individuals experiencing chronic stress exhibit elevated cortisol levels, decreased brain-derived neurotrophic factor (BDNF) and tyrosine receptor kinase B (TrkB) expression, and impaired synaptic plasticity [19,20]. These are mechanisms that may also be involved in the pathophysiology of OTS, and mood disturbances. TrkB is the receptor for BDNF and is involved in the regulation of neurogenesis [21].

Chronic stress not only disrupts neurotrophin expression but also leads to hippocampal dopamine receptor (DR) dysregulation, specifically affecting D1R and D2R, each with distinct functional consequences. D1R dysregulation compromises synaptic plasticity and impairs spatial memory formation [22,23], while D2R dysfunction disrupts emotional regulation and reduces stress resilience [24,25]. Additionally, chronic stress downregulates the 5-hydroxytryptamine receptor (5-HT1A), which is also known as the serotonin receptor, which contributes to anxiety-like behavior and cognitive impairment [26]. A reduction in 5-HT1A expression is also thought to be driven by prolonged corticosterone elevation [27]. Collectively, these disruptions likely contribute to the cognitive impairments and mood disturbances observed in OTS.

Given the well-documented impact of OTS on neurobiological function, including neurotransmitter imbalances, chronic stress, and impaired neuroplasticity, potential therapeutic interventions remain largely unexplored. Essential amino acids (EAA) are widely supplemented by competitive athletes to increase muscle protein synthesis and enhance recovery from exercise [28]. Although EAA have been extensively studied for their role in skeletal muscle repair, significantly less is known about their potential impact on brain function, neuroplasticity, and cognitive resilience, particularly in athletes experiencing OTS. A recent study suggested that EAA supplementation may help restore neurotransmitter homeostasis and mitigate stress-induced cognitive impairments [29]. Investigating whether EAA supplementation can mitigate OTS-related neurobiological disturbances represents a promising yet underexplored approach to optimizing both cognitive resilience and overall recovery. Thus, the purpose of this study was to examine whether EAA supplementation can mitigate neurobiological disturbances and behavior associated with OTS in a resistance training model for mice.

## 2. Materials and Methods

### 2.1. Animals

Thirty-two C57Bl/6J mice (Envigo, Jerusalem, Israel), aged three months, were acclimated to housing conditions for at least seven days. The mice were housed in groups of five per cage within a temperature-controlled vivarium set at 21 °C, with a reversed 12 h light/dark cycle. Food and water were provided ad libitum. Following acclimation, the mice were randomly assigned to one of four experimental groups (n = 8 per group): Resistance training (RT), resistance training with overtraining (RTO), resistance training with overtraining and EAA administration (RTOEAA), or control (CTL). Mice in the RTOEAA group received intraperitoneal injections of EAA at a dose of 1.5 g·kg·day^−1^ for three consecutive days (days 4–6) during each week of the over-training phase. EAA ingestion was also provided ad libitum in the animals drinking water at 2.5 g per 160 mL The EAA supplementation was a proprietary blend provided by Amino Medical Science (Lewes, DE, USA) and contained leucine, isoleucine, valine, phenylalanine, lysine, threonine, methionine, histidine, and the non-essential amino acid tyrosine. All other groups received phosphate-buffered saline (PBS) injections at the same frequency. This study was performed according to the principles and guidelines of the National Institute of Health Guide for the Care and Use of Laboratory Animals. All treatment and testing procedures were approved by the Animal Care Committee of Ariel University (AU-IL-2409-113).

### 2.2. Experimental Design

**Figure 1:**
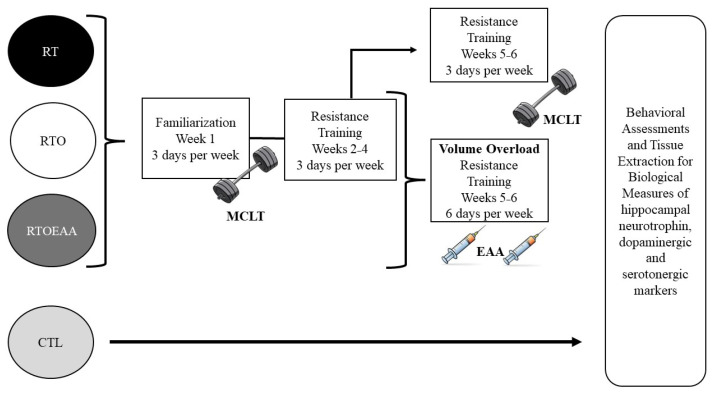
Study Design. RT = resistance training; RTO = resistance training with overtraining; RTOEAA = resistance training with overtraining and EAA administration; CTL = control; EAA = Essential Amino Acids.

The experimental design of this study is depicted in Figure 1. The resistance training protocol was 5 weeks in duration. During the final two weeks of the resistance training protocol the volume of training was increased to cause the OTS. The EAA intervention for the RTOEAA group occurred during the two-week high-volume training period. In consideration that assessment of spatial learning and memory required 5 days of assessment, it was decided a priori to begin initial behavior measures during week three of the exercise protocol. A concern by the investigators was that if the behavior measures would begin following the overtraining period, that recovery from the overtraining stimulus would potentially mask the effects of the EAA intervention. Considering that exercise is known to improve learning outcomes [30], all behavioral measures occurred prior to any resistance exercise session. Spatial learning and behavior assessments were also conducted on the last day of week four and 48 h following the last training session in week 5. Anxiety assessment was conducted 48 h following the last training session.

### 2.3. Resistance Training Protocol

A ladder-climbing exercise model was employed as the resistance for this study. This model required the animals to climb a ladder, 100 cm in length, at an 85° angle with 1.5 cm between each step [31]. The exercise protocol was initiated with a familiarization phase, which was performed without any resistance. During the familiarization phase, the mice climbed the ladder three times, starting from different positions (upper, middle, and base). Between each climb, the mice rested for 60 s. This protocol was performed for five consecutive days. Following the familiarization phase, a maximum load carrying test (MLCT) was conducted to assess the maximum carrying load (MCL) of a single ladder climb as previously suggested [32]. During the MLCT, weight was attached to a small bottle tied to the mouse’s tail. The initial starting weight for the MLCT was 10% of the mouse’s body mass, and after every successful climb, the load was increased by an additional 10% of body mass until failure. A 2 min rest period was provided between each attempt until failure. Failure was recognized as two consecutive unsuccessful attempts to climb the ladder. MCL was identified as the highest load carried.

Following the familiarization week, mice in the RT, RTO and RTOEAA groups performed ladder-based resistance training three times per week, with 48 h between each training session for three weeks. Each session required the animals to perform eight sets of ladder climbs with two minutes of rest between each climb. The protocol required that each mouse perform two sets using a load equal to 50%, 75%, and 85% of their MCL. A single set with 100% of their MCL was then performed. If successful, 10% of their body mass was added to their tail, and the mouse performed an additional climb (a total of 8 maximum climbs were conducted per session). If the mouse completed the climb, that weight would become their new MCL. If unsuccessful, the same resistance was maintained for the next training session. All training sessions were recorded in a logbook. Forty-eight hours after the last training session, a final MCLT was conducted to assess MCL performance. This resistance training protocol was based upon previous work that demonstrated significant increases in muscle mass, muscle fiber cross-sectional area and maximum strength in mice using this protocol [33].

The volume overload to create overtraining was employed during the fourth and fifth weeks of training. Both RTO and RTOEAA groups doubled the training sessions from three days per week with a 48 h recovery period to six consecutive days of training. The exercise protocol for each training session was the same as that used during the previous three weeks. During these sessions if the animals were unable to successfully complete the climb, there was no change in the loading for the following training session. Regardless of their success, all animals attempted 8-sets for each session during the volume overload week. The last successful climb in the last training session was recorded as their final MCL.

### 2.4. Behavioral Measures

Tests of anxiety and spatial memory were performed in a closed, quiet, light-controlled room. Anxiety was assessed using the elevated plus maze (EPM). The EPM was elevated 0.75 m from the floor and consisted of two open arms, two closed arms and a central platform. The closed arms comprised laminated polyvinyl chloride (PVC) material that was 15 cm high. Each arm was 30 cm in length and 5 cm wide. Mice were positioned at the maze center, facing an open arm, and allowed to explore the maze for 5 min. Each session was videotaped and subsequently scored by an independent observer. All results were recorded using an Ethovision automated tracking system (Version 17, Noldus Information Technology, Wageningen, The Netherlands). Arm entry was defined as the animal entering an arm with all four paws. Behaviors assessed included time spent (duration) in the open and closed arms, the frequency of open and enclosed arm entries, and total exploratory activity (entries into all arms). Total exploration was calculated as the number of entries into any arm of the maze to distinguish between impaired exploratory behavior, exploration limited to closed arms (avoidance) and free exploration. After each trial, 70% ethanol was used to disinfect the EPM. An anxiety index was derived from these behavioral measures [34], and was calculated as follows:

$$1-\;\frac{\frac{time\;spent\;in\;the\;open\;arms\;}{total\;time\;on\;the\;maze\;}+\frac{number\;of\;entries\;to\;the\;open\;arms}{total\;exploration\;on\;the\;maze}}{2}$$

Anxiety index values range from 0 to 1, where higher values indicated increased anxiety-like behavior.

Spatial learning and memory were assessed using a modified Barnes maze [35]. The Barnes maze was performed on a 100 cm-diameter circular table with 32 evenly spaced, 5 cm-diameter holes positioned 7.5 cm from the outer edge. The maze was elevated on a pedestal at the center of the room, which contained four distinct visual cues, one on each wall. To facilitate escape box localization, the room was moderately to brightly illuminated (i.e., approximately 1300 lux), encouraging mice to seek the darkened shelter due to their natural aversion to bright, open spaces [35]. An escape box was positioned beneath one of the holes, serving as the goal location. The test was initially conducted during week three of the study, prior to the overtraining phase. All assessments on the Barnes maze occurred prior to any exercise training. During the initial day of the assessment each mouse was familiarized with the escape box for 1 min. The mouse was then placed in the center of the maze and allowed to explore for 2 min or until it entered the escape box. If a mouse failed to locate the escape box, it was gently guided inside and remained there for 20 s. After each trial, 70% ethanol was used to disinfect the table and an escape box was used to minimize olfactory cues. A total of three trials were performed over four days of assessment during week three of the study, prior to the overtraining phase of the study. The Barnes maze assessment was also conducted at the end of each week of the overtraining phase. A total of six assessments were conducted. Spatial memory was determined by examining the average latency time to reach the escape box over the six trials, and the learning slope was calculated.

### 2.5. Tissue Preparation

Twenty-four hours after the final assessment, mice were deeply anesthetized via an intraperitoneal injection of 400 μL of Pentobarbital sodium (20 mg·mL^−1^), CTS Chemical Industries, (Kiryat Malakhi, Israel) and perfused transcardially with PBS to remove circulating blood and clear tissue for extraction. Following perfusion, the hippocampi were carefully dissected, immediately snap-frozen in liquid nitrogen, and stored at −80 °C until further analysis.

### 2.6. Western Blot Analysis

The hippocampus from each animal was homogenized in RIPA lysis buffer (Merck, Germany) and protease inhibitor cocktail (Merck, Germany) at a 1:100 dilution factor. Protein concentration was evaluated using a BCA kit (Merck, Germany) according to the manufacturer’s instructions. Samples were denatured by adding β-mercaptoethanol (1:10, Merck, Germany) and heated at 95 °C for 5 min. The denatured protein content of the homogenates was separated using sodium dodecyl sulfate polyacrylamide gel electrophoresis (SDS-PAGE). In addition, a pre-stained protein ladder (Abcam, Waltham, MA, USA) was loaded onto the gel to confirm the size of the proteins of interest. Separated proteins were transferred from the gel to a nitrocellulose membrane using a transfer pack (Bio-rad, Hercules, CA, USA) and a turbo transfer device (Bio-rad, CA, USA). After completion of the transfer, the membrane was washed with Ponceau S solution (Abcam, Cambridge, UK) to verify the completeness of the transfer. The membrane was then incubated in a blocking solution with 5% BSA diluted in PBS for 1 h at room temperature. Following blocking incubation, the membrane was incubated overnight at 4 °C in a cocktail of primary antibodies containing PBS with 5% BSA, Mouse anti-GR (1:400, Santa Cruz (TX, USA), sc-393232), Rabbit anti-BDNF (1:1000, Abcam (UK), ab108319), Rabbit anti-TrkB (1:1000, Abcam (UK), ab187041) Rabbit anti-D1R (1:000, Abcam (UK), ab279713), D2 Rabbit anti-D2R (1:500, Abcam (UK), ab85367), Rabbit anti-5-HT1A (1:1000, Abcam (UK), ab259896), and Mouse anti-α tubulin (1:1000, Santa Cruz (TX, USA), sc-5286). On the second day, the membrane was washed three times for 5 min with tris buffer saline-tween (TBST) (Bio-Lab, Israel) before being incubated for 1 h at room temperature in a cocktail of fluorescent secondary antibodies containing PBS with 5% BSA, anti-mouse Alexa Fluor^®^ 680 (Jackson ImmunoResearch (West Grove, PA, USA), 711-655-152) diluted 1:15,000, and anti-rabbit Alexa Fluor^®^ 790 (Jackson ImmunoResearch (PA, USA), 715-625-150) diluted 1:15,000. The membrane was rewashed as described and scanned for protein visualization and analysis using the Odyssey CLx system (LI-COR, Lincoln, NE, USA) resolution: 169 μm; intensity: auto mode; according to the manufacturer’s instructions. The fluorescence intensity of proteins of interest bands were determined using the Odyssey Infrared Imaging System software (Image StudioV5.2, Li-Cor Biosciences, Lincoln, NE, USA). Protein expression was calculated as a ratio of the α-tubulin fluorescence intensity.

### 2.7. Statistical Analysis

A Shapiro–Wilk test was used for testing normality. For all performance and biological measures, a one-way analysis of variance (ANOVA) was used to compare performance results between RT, RTO and RTOEAA, and for all molecular results between RT, RTO, RTOEAA and CTL. In the event of a significant F ratio, a Tukey post hoc analysis was used for pairwise comparisons. Due to the novel nature of the animal model of overtraining used in this study, an a priori sample size calculation was not performed. Bivariate correlations between selected measures were performed using Pearson product moment correlations. Outliers were assessed (1.5. interquartile range) and winsorized to the next less extreme value [36]. All statistical analyses were analyzed using SPSS v29 software (SPSS Inc., Chicago, IL, USA), and an α level of *p* ≤ 0.05 was used to determine statistical significance. Performance and body mass data are reported as mean ± SD, while all biological data are reported as mean ± SEM. Effect size was determined using eta squared for all ANOVA measurements.

## 3. Results

### 3.1. Performance and Body Mass

A significant difference (F = 77.13, *p* < 0.001, η^2^ = 0.379) in the average weekly training volume among the three resistance training groups during the five-week training protocol was observed. The average weekly training volume for RTO (1439.2 ± 84.3 g) and RTOEAA (1475.7 ± 174.1 g) were significantly greater than RT (829.9 ± 59.4 g) (*p*’s < 0.001, respectively). The greater training loads for RTO and RTOEAA compared to RT reflected the doubling of the training days from three to six per week during weeks four and five of the training protocol. Examination of the change in MCL from the end of the third week of training to the end of the fifth week of training revealed a significant difference (F = 6.407, *p* = 0.007). Significant differences (*p* = 0.002) in the delta (∆) score (MCL at end of week 3–MCL at end of week 5) were noted between RT (−1.0 ± 4.8 g) and RTO (−12.6 ± 9.0 g). In addition, a significant difference (*p* = 0.029) was also noted between RTO and RTOEAA (−4.9 ± 5.2 g). No significant difference (*p* = 0.254) in ∆MCL was observed between RT and RTOEAA.

Significant differences (F = 3.155, *p* = 0.041) in ∆ (week 5–week 1) body mass was also observed. The ∆ body mass for RTO (−0.56 ± 0.94 g) was significantly different (*p* = 0.005) than CTL (0.63 ± 0.75 g), but not different (*p* > 0.05) compared to RT (−0.06 ± 0.94 g) or RTOEAA (0.08 ± 0.34 g). No other between-group differences were noted.

### 3.2. Behavior Measures

**Figure 2:**
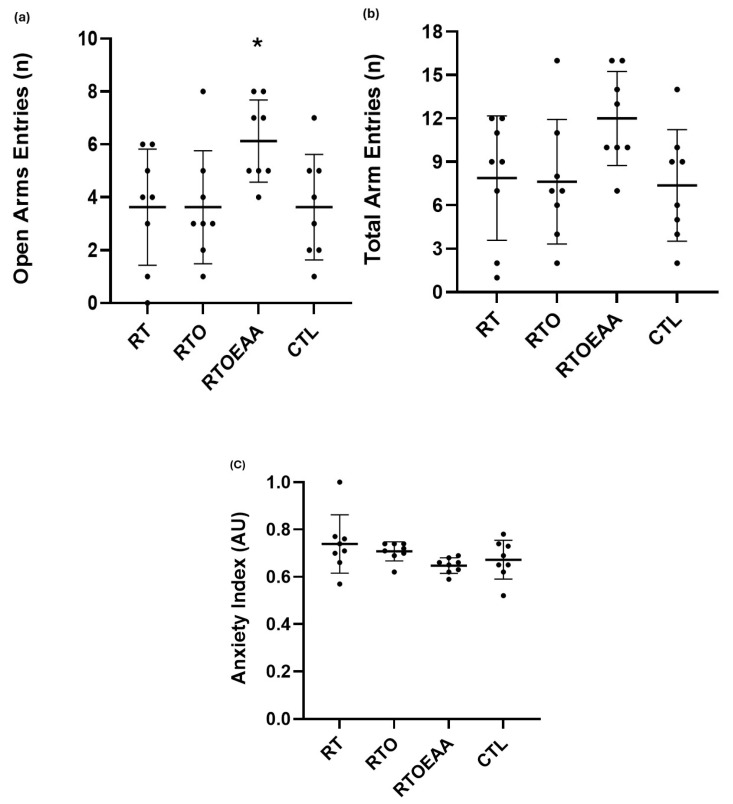
Elevated Plus Maze Comparisons of (**a**) Time Spent in The Open Arms, (**b**) Total Arm Entries, and (**c**) Anxiety Index. RT = resistance training; RTO = resistance training with overtraining; RTOEAA = resistance training with overtraining and EAA administration; CTL = control; * = Significantly greater than all other groups. Data are reported mean ± SD.

Differences between the groups in the time spent in the open arms, total arm entries and anxiety index can be observed in Figure 2a–c, respectively. One-way ANOVA revealed a significant difference (F = 3.167, *p* = 0.040, η^2^ = 0.243) between the groups in open arm entries, but no differences were noted in total arm entries (F = 2.476, *p* = 0.082, η^2^ = 0.202) and anxiety index (F = 2.118, *p* = 0.120, η^2^ = 0.182). Post hoc analysis indicated that the number of open arm entries for RTOEAA were significantly greater (*p* < 0.05) than all other groups.

**Figure 3:**
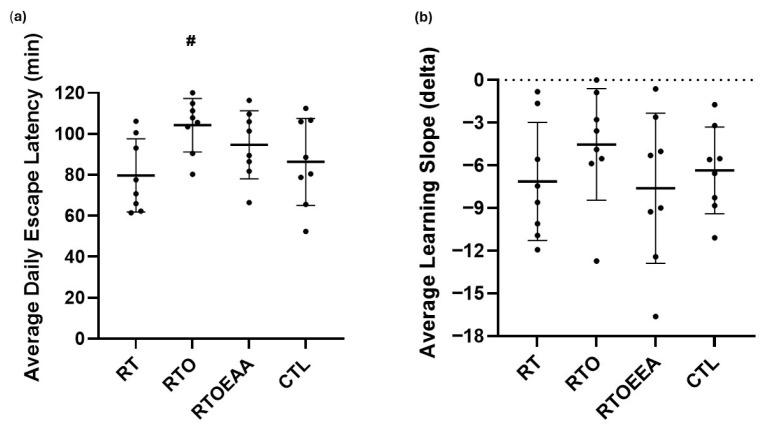
Barnes Maze Comparison of (**a**) Average Daily Escape Latency and (**b**) Learning Slope. RT = resistance training; RTO = resistance training with overtraining; RTOEAA = resistance training with overtraining and EAA administration; CTL = control; # = Significantly greater than RT and CTL. Data are reported mean ± SD.

The average daily escape latency and learning slope for each group during the acquisition phase of the Barnes maze assessment is depicted in Figure 3a and Figure 3b, respectively. A significant difference (F = 2.966, *p* = 0.049, η^2^ = 0.241) was observed in average daily latency, but not in the learning slope (F = 0.838, *p* = 0.484, η^2^ = 0.082). The average daily latency was significantly greater for RTO compared to both RT (*p* = 0.009) and CTL (*p* = 0.05). No other significant differences were noted. Training volume was significantly correlated to average escape latency (r = 0.517, *p* = 0.010). The greater the training volume the longer the latency time was noted.

**Figure 4:**
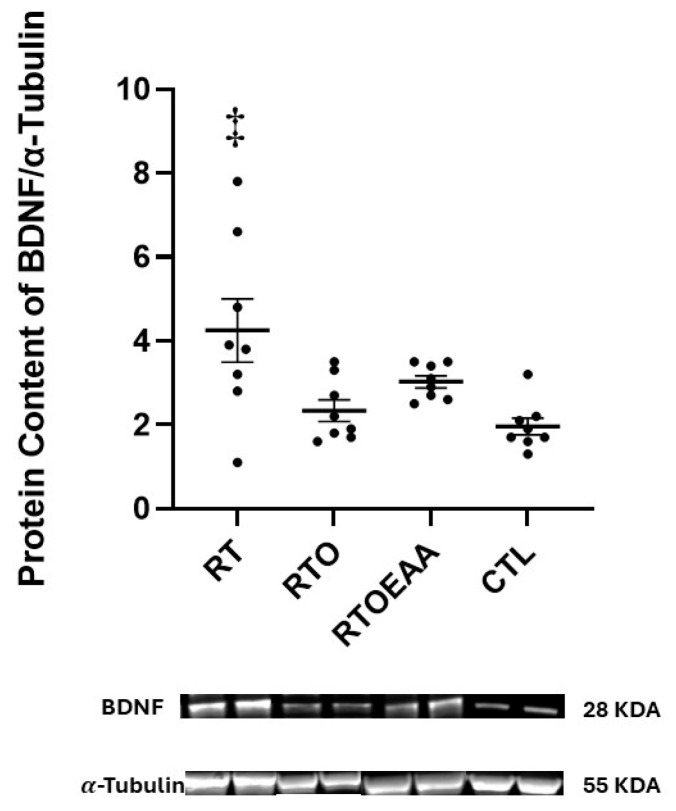
Brain Derived Neurotrophic Factor (BDNF) Expression. RT = resistance training; RTO = resistance training with overtraining; RTOEAA = resistance training with overtraining and EAA administration; CTL = control; ‡ = Significantly greater than RTO and CTL. Representative Western blot images are shown below the quantified data. Individual animal responses and group mean ± SEM are reported.

**Figure 5:**
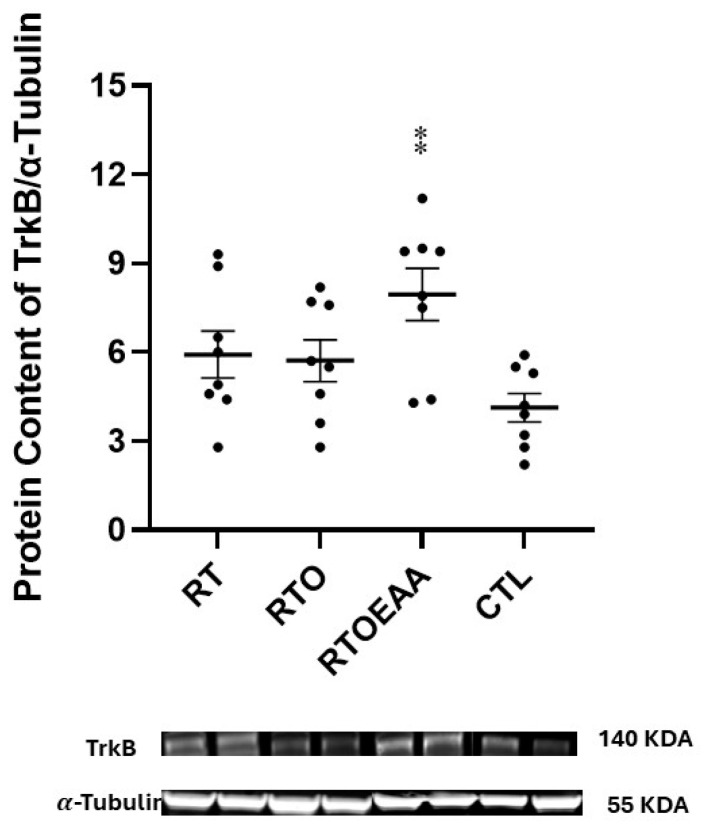
Tyrosine Receptor Kinase B (TrkB) Expression. RT = resistance training; RTO = resistance training with overtraining; RTOEAA = resistance training with overtraining and EAA administration; CTL = control; ⁑ = Significantly different than CTL. Representative Western blot images are shown below the quantified data. Individual animal responses and group mean ± SEM are reported.

**Figure 6:**
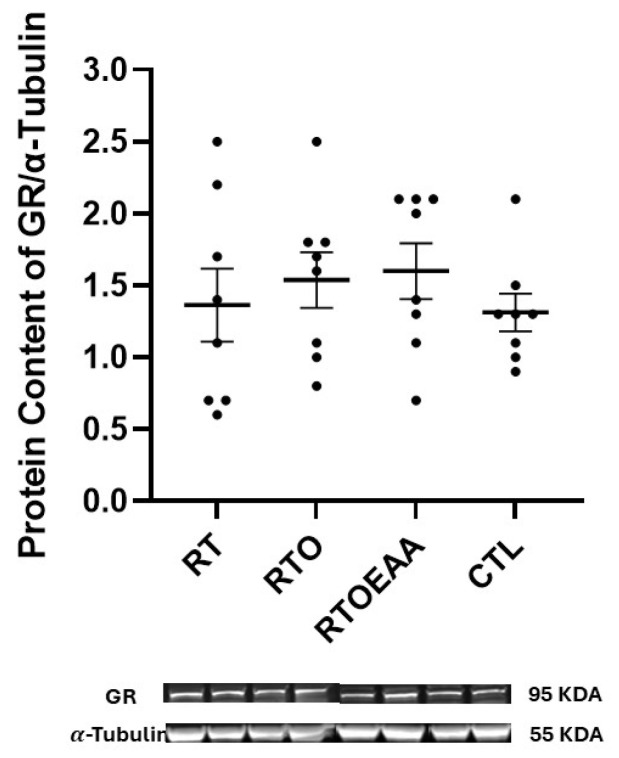
Glucocorticoid Receptor (GR) Expression. RT = resistance training; RTO = resistance training with overtraining; RTOEAA = resistance training with overtraining and EAA administration; CTL = control. Representative Western blot images are shown below the quantified data. Individual animal responses and group mean ± SEM are reported.

Expressions of BDNF, TrkB and GR in the hippocampus can be observed in Figure 4, Figure 5 and Figure 6, respectively. A significant interaction (F = 5.773, *p* = 0.003, η^2^ = 0.382) was noted in BDNF expression. BDNF expression in RT was significantly greater than both RTO (*p* = 0.016) and CTL (*p* = 0.003). No other significant between group comparisons were noted in BDNF expression. A significant interaction (F = 4.615, *p* = 0.010, η^2^ = 0.331) was also noted in TrkB expression in the hippocampus. TrkB expression was significantly greater for RTOEAA compared to CTL (*p* = 0.005). No other significant between group comparisons were observed in TrkB expression. No significant differences (F = 0.479, *p* = 0.699, η^2^ = 0.049) were noted in GR expression. Training volume was inversely correlated with BDNF expression (r = −0.472, *p* = 0.020). These results indicate that elevations in training volume were associated with a down-regulation of BDNF expression in the hippocampus.

**Figure 7:**
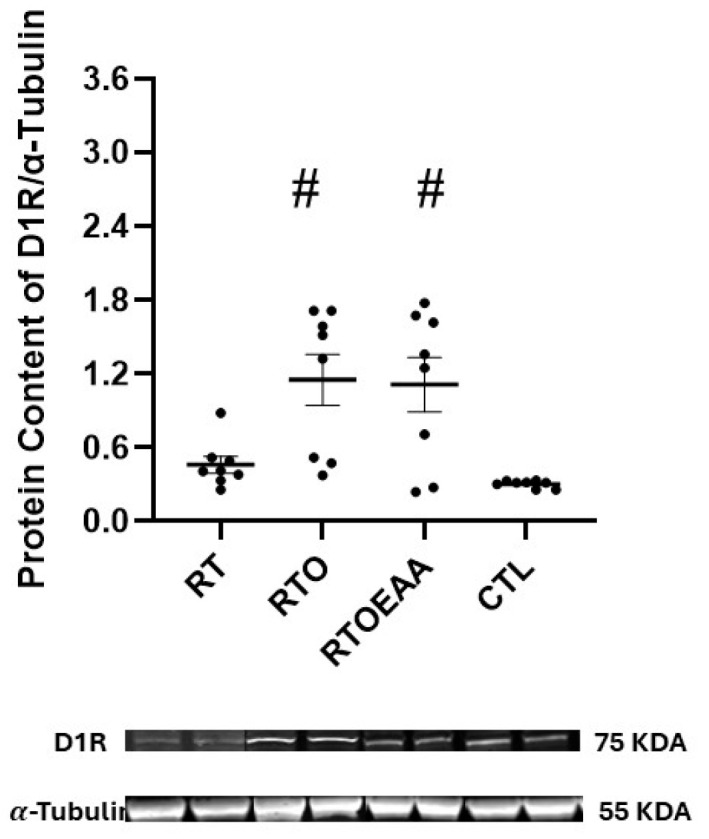
Dopamine 1 Receptor (D1R) Expression. RT = resistance training; RTO = resistance training with overtraining; RTOEAA = resistance training with overtraining and EAA administration; CTL = control; # = Significantly greater than RT and CTL. Representative Western blot images are shown below the quantified data. Individual animal responses and group mean ± SEM are reported.

**Figure 8:**
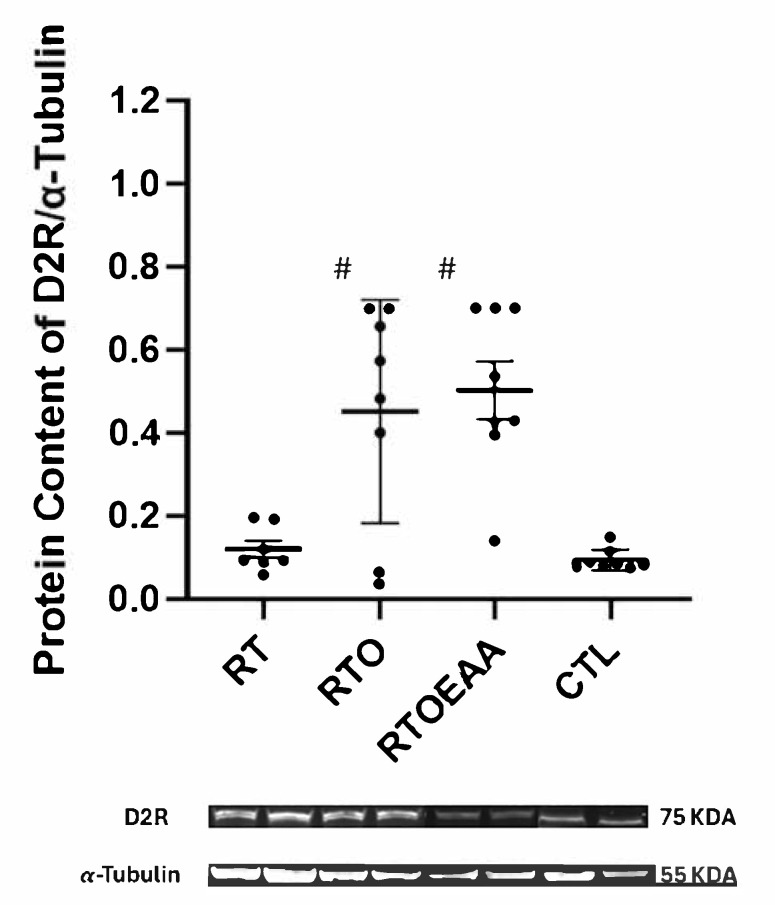
Dopamine 2 Receptor (D2R) Expression. RT = resistance training; RTO = resistance training with overtraining; RTOEAA = resistance training with overtraining and EAA administration; CTL = control; # = Significantly greater than RT and CTL. Representative Western blot images are shown below the quantified data. Individual animal responses and group mean ± SEM are reported.

**Figure 9:**
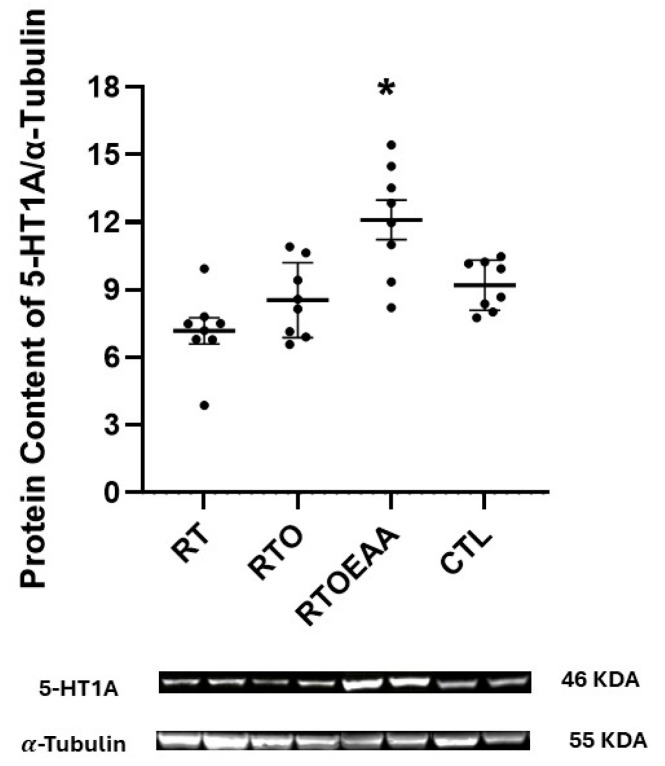
Serotonin Receptor (5-HT1A) Expression. RT = resistance training; RTO = resistance training with overtraining; RTOEAA = resistance training with overtraining and EAA administration; CTL = control; * = Significantly greater than all other groups. Representative Western blot images are shown below the quantified data. Individual animal responses and group mean ± SEM are reported.

Expressions of hippocampal D1R, D2R and 5-HT1A are depicted in Figure 7, Figure 8 and Figure 9, respectively. The one-way ANOVA revealed a significant difference in D1R (F = 7.906, *p* < 0.001, η^2^ = 0.459), D2R (F = 10.816, *p* < 0.001, η^2^ = 0.546) and 5-HT1A (F = 10.645, *p* < 0.001, η^2^ = 0.533) expressions in the hippocampus. D1R expression in the hippocampus was significantly greater for RTO and RTOEAA compared to RT (*p* = 0.019 and *p* = 0.031, respectively) and CTL (*p* = 0.003 and *p* = 0.006, respectively). No other between-group differences were observed for D1R expression. D2R expression was significantly greater for RTO and RTOEAA than both RT (*p* = 0.025 and *p* < 0.001, respectively) and CTL (*p* = 0.013 and *p* < 0.001, respectively). No difference in D2R expression was noted (*p* = 0.490) between RTO and RTOEAA. Post hoc analysis revealed that 5-HT1A expression in RTOEAA was significantly greater than RT (*p* < 0.001), RTO (*p* = 0.003) and CTL (*p* = 0.016). No other between group differences for 5-HT1A were observed. Expressions of D1R, D2R and 5-HT1A were also significantly correlated to training volume (r’s = 0.573, 0.555, and 0.538 with *p* values of 0.003, 0.006, and 0.007, respectively). These results suggest that increases in training volume were associated with elevations in the serotonin-dopamine axis. In addition, elevations in 5-HT1A were inversely correlated with anxiety index (r = −0.404, *p* = 0.022) and significantly correlated with open arm entries in the EPM (r = 0.582, *p* < 0.001).

## 4. Discussion

The purpose of this study was to examine the efficacy of EAA supplementation on increasing resilience to an overtraining stress. This was accomplished by increasing the training frequency during weeks four and five of the study, which resulted in significant increases in training volume for animals in the RTO and RTOEAA groups. Symptoms of the overtraining syndrome are reported to be quite varied and not consistent [37]. The one variable that is consistent for determining that overtraining has occurred is that decrements in physical performance must be seen [37]. The significant decrease observed in ∆MCL of RTO compared to RT provided clear evidence that the animal model employed to stimulate overtraining (e.g., volume overload stress) was effective in its intended goal. In addition, the results of this study also indicated that EAA intervention appeared to increase resiliency to the volume-overload stress as reflected by the significant greater performance outcomes of RTOEAA compared to RTO, and the lack of any differences observed between RT and RTOEAA despite significant differences between the two groups in volume of training completed. In addition, the body mass of the RTO group was significantly lower than CTL group with no other in-between group differences noted suggesting a catabolic effect in the RTO group due to the overtraining stress.

The overtraining protocol also had significant impact on behavioral performance. The animals that were overtrained, and not provided with EAA, experienced greater latency times during the Barnes maze, indicating that the overtraining stimulus negatively impacted spatial memory performance. This is consistent with other animal studies that reported significant declines in memory to an overtraining stimulus [38,39]. These effects were likely the result of changes observed in the neurotrophin response to the overtraining stimulus. Despite changes in memory, the overtraining stimulus did not impair anxiety in the overtrained animals. Interestingly, the animals that were provided EAA supplementation exhibited significantly greater increases in open-arm entries than all other groups, suggesting that the EAA supplementation provided an anxiolytic effect in the overtrained animals. This is consistent with other investigations reporting reductions in depression in both young and older adults supplemented with EAA [40,41]. It is likely that these effects were related to the changes observed for RTOEAA in the dopaminergic and serotonergic pathways.

BDNF has been demonstrated to have an important role in neuronal remodeling and modulating synaptic plasticity [42], and its expression has also been associated with the process of memory consolidation [43]. Decreases in BDNF expression are sensitive to various physical and emotional stresses [44,45] but are elevated by resistance training [46,47]. The results of the present study are consistent with these previous findings. The significant elevation of BDNF expression between RT and CTL is consistent with the positive effect that resistance training has on stimulating BDNF expression. The results of this study are also consistent with existing knowledge on the effect of stress on BDNF expression. The increased training volume used to create the overtraining stress resulted in a significant decline in hippocampal BDNF expression in RTO compared to RT. Moreover, the negative correlation observed between training volume and BDNF expression in the hippocampus was suggestive of a workload-dependent downregulation of BDNF. This is consistent with other studies reporting similar reductions in hippocampal BDNF expression during OTS, albeit induced by endurance training [48,49]. The lower BDNF expression in RTO also appeared to impair spatial memory, as mice in RTO exhibited longer escape latencies compared to RT and CTL. These behavioral outcomes and downregulation of BDNF associated with OTS are consistent with previous studies examining the effect of stress, neurotrophin expression and spatial memory [44,45,48,49].

EAA supplementation appeared to provide some protection to BDNF expression as no statistical difference was noted between RT and RTOEAA. There is only one other study known that has examined EAA supplementation and circulating BDNF concentrations. Dora and colleagues [50] examined the acute effect of EAA supplementation in young adult men that were provided with the supplement 30 min prior to a 30 min bout of endurance exercise. The investigators reported significant improvements in executive function following the EAA trial compared to placebo. However, no changes in circulating BDNF concentrations were noted. No other studies appear to have been conducted either in human or animal models.

TrkB expression did not appear to be affected by the overtraining stimulus. The only difference in TrkB expression noted was between RTOEAA and CTL. The significantly greater TrkB expression in RTOEAA suggested that EAA supplementation and resistance exercise was able to up-regulate TrkB expression in the hippocampus compared to the other groups. Interestingly, TrkB expression in the RT group was not significantly different from CTL, consistent with previous studies indicating limited hippocampal TrkB responsiveness to resistance training in rodents [51,52]. The decline in BDNF expression observed in the RTO group, without any change in TrkB receptor signaling, may indicate that the overtraining stimulus resulted in a greater sensitivity of upstream molecular mechanisms involved in neurotrophin activity. Given the improved spatial performance observed in RTOEAA mice, the elevated TrkB expression may reflect a compensatory neuroprotective response facilitated by EAA supplementation during the training stress. The potential mechanism for how EAA can manipulate TrkB expression is not well understood, as no previous research is known that has examined this specific question.

The volume-overload did not appear to affect hippocampal GR expression. This is consistent with other investigations that have reported no changes in hippocampal GR expression following an overtraining stimulus, albeit using endurance exercise as the training stress [39]. It is possible that the relatively short duration of the daily training stress may not have been of sufficient duration to elevate hippocampal GR expression. In some chronic stress models lasting between 10 days to three weeks, a restraint stress performed for six hours daily resulted in significant elevations in hippocampal GR [53,54]. The volume-overload stress employed in this study was a physical stressor involving high volume exercise, and not a psychological stressor similar to that employed by these former studies. Various forms of stress have a varied effect on the stress response in the brain [55]. The resistance training volume-overload stress employed in this study did not appear to result in a significant hippocampal GR response.

The dopaminergic and serotoninergic systems have been investigated with great interest regarding stress regulation [56,57]. To date, there have been only limited examinations on the effect of OTS on the dopaminergic and serotonergic systems. The volume-overload stress used in this study significantly increased hippocampal D1R expression. This is consistent with previous reports reporting elevated D1R expression following chronic stress [58,59]. The elevation in D1R expression in those studies was also associated with impaired spatial memory. The significant correlation observed between training volume and D1R expression in this study is suggestive of a dopaminergic receptor maladaptation in response to an excessive training stress. The increased escape latency observed in the RTO group likely reflected the impaired spatial memory, which likely was mediated by heightened D1R signaling. This maladaptive response underscores the potential negative consequences of OTS on hippocampal dopaminergic signaling and related cognitive performance.

D2R expression in the hippocampus is thought to be involved in regulating memory processes, mood, and stress resilience [24,25,60]. Increases in depressive-like behavior has been shown to be associated with decreases in D2R expression in mice [61]. Although the overtraining stimulus did not appear to affect D2R expression, as no differences were noted between RT and RTO, training volume was significantly correlated with hippocampal D2R expression. This is supported by other investigations that have demonstrated that exercise, albeit endurance exercise, can increase D2R expression [62,63]. Dopamine synthesis is dependent upon the non-essential amino acid tyrosine and phenylalanine [64]. Both amino acids were consumed by RTOEAA. Whether essential amino acids can stimulate an upregulation of dopamine receptors is not well-understood. Interestingly, branched-chain amino acids (BCAA) are known to reduce tyrosine uptake into the brain, subsequently lowering dopamine synthesis [63]. By providing tyrosine to a BCAA supplement, this detrimental effect on dopamine synthesis may be avoided [64]. The results of the present study suggest that high volume resistance exercise may be a more potent stimulus than EAA supplementation that contains both phenylalanine and tyrosine on increasing D2R.

The 5-HT1A receptor within the hippocampus is a known regulator of anxiety and depression during chronic stress [56,57]. Decreases in 5-HT1A are associated with deficits in performance on spatial memory tasks, while upregulation of the receptor results in improved spatial memory performance [65]. Similarly, deficits in 5-HT1A receptor expression are also associated with increased anxiety, which is enhanced when 5-HT1A receptors are returned to normal levels [66]. The significant correlation observed between hippocampal 5-HT1A expression and open arm entries in the EPM, and the significant inverse correlation between 5-HT1A and anxiety index are supportive of the anxiolytic effect associated with an elevation of 5-HT1A expression.

The volume-overload stress employed in this study, designed to create the overtraining stimulus, did not appear to affect 5-HT1A expression, as no statistical difference was noted between RTO and RT. These results appear to be supportive of previous research that reported no change in brain serotonin transporter binding reuptake in overtrained men and women athletes [67]. The hypothesis that hippocampal 5-HT1A expression may be affected by overtraining was based on a previous case study that reported abnormal serotonin uptake values in an overtrained athlete [68], and other studies that demonstrated decreased 5-HT1A expression during chronic stress models [27]. It is possible that the volume-overload stress used in this study was not of sufficient duration to impact the serotonergic pathway. More so, training volume was significantly correlated to 5-HT1A expression suggesting that the volume-overload stress may have provided a greater stimulus for 5-HT1A expression, and potentially indicates a sensitive compensatory response to acute changes in training volume. Perhaps these results are indicative that the OTS observed in this study was more related to nonfunctional overreaching than full-blown overtraining [37]. The difference between the two would be the extent of recovery required to return to baseline performance.

The significantly greater 5-HT1A expression in RTOEAA compared to all other groups suggests that the EAA supplement stimulated 5-HT1A up-regulation. Previous research has suggested that running exercise combined with BCAA supplementation may reduce brain serotonin levels due to an oversaturation of the amino acid transporter that can limit tryptophan uptake, the precursor for serotonin synthesis [69]. It has been suggested that increases in circulating concentrations of BCAA and other EAAs result in a greater EAA/tryptophan plasma ratio that can reduce brain serotonin synthesis [67]. This has been supported by other investigations that have also demonstrated significant declines in serotonin levels following acute BCAA ingestion [64,70,71]. To the best of our knowledge, this is the first study to examine the effect of an EAA supplementation on hippocampal 5-HT1A expression. Although speculative, it is possible that the EAA supplementation did reduce hippocampal serotonin levels, but the compensatory response was an upregulation in the serotonin receptor. There is limited evidence that has reported regional patterns of compensatory blunted serotonergic regulation [72]. Unfortunately, hippocampal serotonin levels were not examined in the present study, which limits our discussion of this physiological effect.

There are limitations in this study that also need to be acknowledged. This study used a novel, but specific, resistance training volume overload to stimulate the overtraining response. Physiological alterations due to overtraining are often specific to the type of activity performed (e.g., endurance versus strength/power). Thus, the results of this study may not be applicable to overtraining models during endurance performance. In addition, the use of only two behavioral measures, the Barnes maze to examine spatial memory and the EPM to examine anxiety, may not provide firm conclusions on the effect of overtraining and behavior. The use of additional complimentary assessments may have provided greater confirmation of the results. However, additional behavioral measures would have required additional days of testing, which may have changed the outcome as recovery from the overtraining paradigm may have resulted.

## 5. Conclusions

In summary, the overtraining model incorporated into this study resulted in significant changes in muscle performance and behavior. Specifically, significant declines in ∆MCL were noted in RTO compared to RT and RTOEAA, confirming that the animals in that group were overtrained. Behaviorally, changes in the average latency times for RTO indicated that the overtraining protocol also resulted in spatial memory deficits. These latter changes may have been related to the significant declines in BDNF expression and elevations in D1R expressions. This study also demonstrated that EAA supplementation appeared to increase resiliency to the volume-overload stress, possibly by stimulating significant increases in hippocampal TrkB and 5-HT1A expression. In conclusion, EAA supplementation during a resistance model of overtraining appeared to provide increased resiliency to the volume-overload stress by maintaining neurotrophin expression and enhancing serotonergic adaptation.

## Data Availability

The data presented in this study are available on request from the corresponding author.
